# Regulated Cell Death in Traumatic Brain Injury: Investigating Mechanisms Contributing to Cognitive Impairment

**DOI:** 10.3390/cells14231878

**Published:** 2025-11-27

**Authors:** Yu Xia, Mengzhu Li, Zhenhuan Chen, Mingbo Fan, Qihang Pan, Yahui Tian, Xiaolong Liu, Pengcheng Du, Jun Li

**Affiliations:** 1 Department of Neurosurgery, The Central Hospital of Wuhan, Tongji Medical College, Huazhong University of Science and Technology, Wuhan 430014, China; 2 Key Laboratory for Molecular Diagnosis of Hubei Province, The Central Hospital of Wuhan, Tongji Medical College, Huazhong University of Science and Technology, Wuhan 430014, China; 3 Department of Neurosurgery, Maternal and Child Health Hospital of Hubei Province, Tongji Medical College, Huazhong University of Science and Technology, Wuhan 430070, China; 4 Department of Neurosurgery, People’ s Hospital of Dongxihu District, Wuhan 430040, China

**Keywords:** traumatic brain injury, cognitive impairment, apoptosis, necroptosis, pyroptosis, ferroptosis, cuproptosis

## Abstract

Cognitive impairment remains one of the most prevalent and debilitating sequelae of traumatic brain injury (TBI), profoundly compromising long-term quality of life. Nevertheless, effective treatment options are limited, as the complexity of post-TBI pathology often exceeds the protective scope of conventional neuroprotective strategies. Accumulating research has revealed regulated cell death (RCD) as a central driver of neuronal loss and cognitive decline post-TBI. Consequently, targeting RCD pathways has emerged as a promising strategic direction for alleviating post-TBI cognitive impairment. This review provides an analysis of the molecular mechanisms underlying five major RCD forms, including apoptosis, necroptosis, pyroptosis, ferroptosis, and cuproptosis. Furthermore, it critically assesses the therapeutic potential of these pathways while examining their complex interplay in post-TBI cognitive impairment. By systematically synthesizing recent advances in targeted therapeutic strategies, we highlight that targeting RCD pathways paves the way for highly effective and precise therapeutic modalities against post-TBI cognitive impairment, although challenges in multi-target combination therapies and brain delivery warrant further investigation.

## 1. Introduction

Traumatic brain injury (TBI) is defined by the Centers for Disease Control and Prevention (CDC) as a disruption in normal brain function resulting from an external mechanical force to the head, such as a blow, jolt, or penetrating injury [1]. Globally, an estimated 69 million individuals sustain a TBI annually, with falls and road traffic incidents constituting the major etiologies [2].

The pathophysiology of TBI comprises two distinct phases: primary and secondary injury. Primary injury occurs instantaneously at the moment of impact, causing mechanical damage to neurons and vasculature. This leads to direct cell death and activates inflammatory pathways [3]. Secondary injury begins within hours and continues over days to weeks, driven by molecular cascades such as glutamate excitotoxicity, oxidative stress, and mitochondrial dysfunction, all of which exacerbate neuronal loss through regulated cell death (RCD) processes [4,5,6]. Unlike the uncontrolled accidental cell death from direct trauma, RCD is a genetically programmed process that eliminates compromised cells [7]. Key RCD modalities, including apoptosis, necroptosis, pyroptosis, ferroptosis, and cuproptosis, contribute significantly to secondary injury [8]. The activation of these pathways causes delayed and often irreversible neuronal loss in brain regions critical for cognition, establishing RCD as a key driver of the long-term cognitive deficits associated with TBI [9].

Among the various sequelae that arise following secondary brain injury, cognitive impairment is one of the most prevalent and debilitating outcomes [10]. It typically manifests as deficits in multiple domains, including language ability, logical reasoning, and executive control, substantially compromising patients’ daily functioning and quality of life [11]. Given the restricted efficacy of existing interventions, which predominantly involve conventional neuroprotective approaches, developing effective treatments for TBI-related sequelae represents a critical unmet need [12]. Therefore, exploring therapeutic strategies that target the mechanisms of RCD, a key driver of secondary injury, holds significant clinical potential for mitigating post-TBI cognitive impairment and merits intensified research.

This review analyzes the molecular mechanisms of RCD in post-TBI cognitive impairment. By integrating this analysis with emerging therapeutic targets and pathway interactions, and considering translational challenges like human TBI heterogeneity, we ultimately aim to propose novel interventions for patients.

## 2. Molecular Mechanism of Apoptosis

In 1972, Kerr et al. first introduced the concept of apoptosis, a process morphologically characterized by cell shrinkage, nuclear condensation, DNA fragmentation, and the formation of apoptotic bodies under an intact plasma membrane [13]. Apoptotic execution requires the activation of caspases via two distinct pathways: the intrinsic pathway and the extrinsic pathway [14]. On the one hand, the intrinsic apoptotic pathway is initiated in response to diverse intracellular stresses, such as toxic insults, DNA damage, or endoplasmic reticulum stress [15], which promote the competitive binding of BH3-only proteins to antiapoptotic Bcl-2 family members, leading to the liberation and activation of the proapoptotic effectors BAX and BAK. Thereafter, oligomerized BAX/BAK enhances mitochondrial outer membrane permeability (MOMP), releasing cytochrome c into the cytosol [16,17,18]. The released cytochrome c facilitates the assembly of the apoptosome by binding to apoptosis protease activating factor-1 (Apaf-1), which activates initiator caspase-9 and subsequently cleaves effector caspase-3, thereby initiating the apoptotic cascade [19]. A caspase-independent intrinsic apoptotic pathway has been identified, which is mediated by apoptosis-inducing factor (AIF) [20]. Upon proteolytic cleavage, AIF translocates from the mitochondrial inner membrane to the nucleus, where it directly induces chromatin fragmentation [21]. On the other hand, the extrinsic apoptotic pathway is triggered by the binding of extracellular ligands, such as FasL, to transmembrane death receptors like Fas [22,23]. This interaction induces the assembly of the death-inducing signaling complex (DISC), leading to the activation of the initiator caspase, caspase-8 [24]. Subsequently, activated caspase-8 proteolytically cleaves and activates the executioner caspase-3, ultimately culminating in apoptosis [25].

## 3. Therapeutic Potential of Apoptosis in Post-TBI Cognitive Impairment

As early as 1997, Yakovlev et al. identified apoptosis as a critical pathological process underlying cognitive impairment following TBI [26]. In their model, DNA fragmentation, a biochemical hallmark of apoptosis, was detected, along with a significant increase in both mRNA expression and enzymatic activity of caspase-3 (a central apoptotic protease [27]) in the lateral cortex and hippocampus following TBI [26]. These findings indicate that apoptosis, observable even after mild TBI, leads to neuronal loss within cognition-critical regions, thereby directly compromising neurological function [28]. Accordingly, a study has demonstrated that caspase-3 mediates neuronal death following TBI in rats, and that the caspase-3 inhibitor N-benzyloxycarbonyl-Asp-fluromethyl ketone (z-DEVD-fmk) effectively reduces cerebral tissue damage in TBI models [29].

The translocation of AIF, a key mediator of caspase-independent apoptosis, is triggered by the activation of poly(ADP-ribose) polymerase-1 (PARP-1) [30]. A study demonstrated that administration of the PARP-1 inhibitor INO-1001, an indeno [1,2-c]isoquinolinone derivative, within 24 h post-TBI in mice improved cognitive function. This beneficial effect was attributed to PARP-1 inhibition, which subsequently inhibited the caspase-independent apoptotic pathway mediated by AIF, thereby suggesting AIF as a potential therapeutic target [31]. Additionally, caspase-8 functions as the apical protease of the extrinsic apoptotic route. Clinical research has revealed that TBI patients with high caspase-8 levels showed higher mortality rates [32]. Furthermore, a study also indicated that caspase-8 expression is significantly upregulated in TBI mice, and neuron-specific knockdown of caspase-8 attenuates neuronal apoptosis and improves neurological function in TBI mice [33]. These findings are corroborated by experiments in which pharmacological inhibition of caspase-8 diminished neuronal apoptosis and neurological dysfunction, underscoring its importance in the acute pathophysiology of TBI.

Pharmacological interventions that modulate apoptosis have demonstrated considerable success in ameliorating TBI pathology. Azithromycin exhibits robust neuroprotective effects following TBI by modulating key apoptotic regulators, including the downregulation of caspase-3 and the proapoptotic protein BAX, alongside the upregulation of the antiapoptotic protein Bcl-2, ultimately leading to significant improvements in spatial memory and recognition in rats [34]. Similarly, Lupeol has been shown to suppress reactive oxygen species generation and lipid peroxidation, reduce the expression of proapoptotic factors such as BAX, cytochrome c release, and caspase-3 activation, ultimately leading to the improvement of hippocampal-dependent cognitive function [35]. Edaravone improves TBI-induced learning and memory deficits in rats by inhibiting hippocampal neuronal apoptosis through activation of the BDNF/TrkB pathway [36]. Pifithrin-α and its oxygen analog PFT-α(O) have been shown to ameliorate TBI-induced learning and memory dysfunction in rats by inhibiting p53-dependent apoptosis [37]. Previous study demonstrated that COG1410, a triggering receptor expressed on myeloid cells 2 (TREM2) activator, improves post-TBI cognitive impairment through activation of the Akt/CREB/BDNF pathway and inhibition of caspase-3-dependent neuronal apoptosis [38]. Moreover, a recent study demonstrated that the small-molecule drug ZL006 ameliorates cognitive dysfunction in mice following TBI by inhibiting the interaction between nNOS and PSD95, thereby reducing NO-mediated oxidative damage and suppressing caspase-3-dependent neuronal apoptosis [39]. Recent work revealed that HS38 attenuates neuronal apoptosis and ameliorates cognitive dysfunction in mice following TBI by inhibiting ZIPK activity, thereby reducing DEDD-mediated activation of caspase-3 [40]. Furthermore, several physical interventions have shown promise in modulating apoptotic pathways to improve cognitive outcomes after TBI. Mild hypothermia therapy, for instance, has been found to downregulate caspase-3 and upregulate Bcl-2, thereby attenuating neuronal apoptosis and exerting protective effects against TBI-induced cognitive deficits [41]. Similarly, a study demonstrated that hyperbaric oxygen therapy suppresses cleaved caspase-3 levels, preserves mitochondrial respiratory function, and enhances spatial learning in murine TBI models [42].

Several pharmacological agents targeting apoptotic pathways have advanced to clinical development. Previously, N-methyl-D-aspartate (NMDA) receptor inhibitors have been confirmed to reduce hippocampal caspase-3 expression, mitigate apoptosis, and alleviate cognitive impairment [43]. Clinical evaluation further demonstrated that Traxoprodil improved cognitive function in TBI patients [44]. Experimental models showed that statins modulated the anti/proapoptotic balance and enhanced spatial learning in rodents, while double-blind trials confirmed their efficacy in promoting memory recovery in moderate-to-severe TBI cases [45,46]. Moreover, preclinical evidence indicated that progesterone mitigated apoptosis and supported cognitive recovery, findings subsequently validated in a Phase II randomized controlled trial [47]. These results underscore the translational promise of apoptosis-targeted strategies while highlighting the need for expanded clinical investigation.

**Figure 1:**
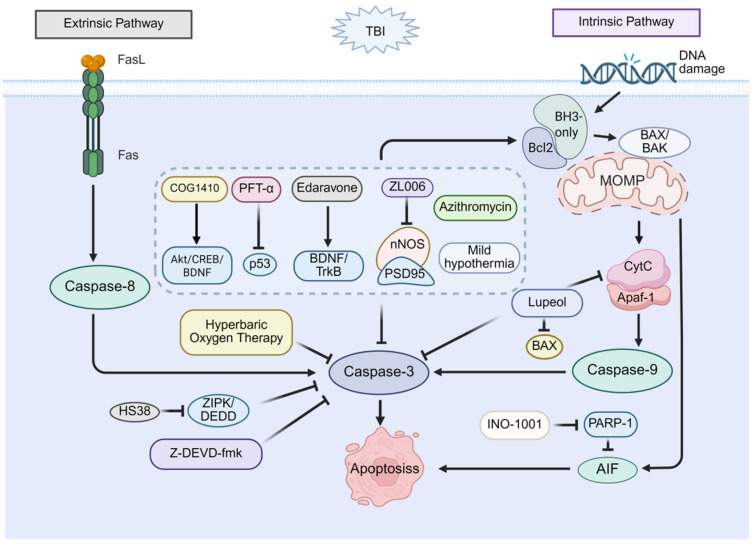
Therapeutic potential of apoptosis in post-TBI cognitive impairment. Apoptosis occurs through two main pathways. The intrinsic pathway initiates when BH3-only proteins bind and inhibit Bcl-2, leading to BAX activation, mitochondrial outer membrane permeability (MOMP), cytochrome c release, apoptosome formation, and subsequent activation of caspase-9 and caspase-3. The extrinsic pathway begins with FasL-Fas binding, activating caspase-8, and directly cleaving caspase-3. Pharmacological inhibition strategies include: agents that inhibit caspase-3, upregulate Bcl-2, and downregulate BAX (COG1410 via Akt/CREB/BDNF; PFT-α via p53 inhibition; Edaravone via BDNF/TrkB signaling; ZL006 via nNOS-PSD95 disruption; Azithromycin; Mild hypothermia); targeting caspase-3 inhibition (z-DEVD-fmk; HS38 via ZIPK/DEDD; Hyperbaric Oxygen Therapy); and other multi-target agents (Lupeol, which inhibits caspase-3, downregulates BAX, and reduces cytochrome c release; INO-1001, which inhibits PARP-1/AIF). Together, these strategies reduce neuronal death and cognitive impairment.

Collectively, these studies demonstrate that targeted inhibition of apoptotic pathways, whether directed against executioner caspases (such as caspase-3), initiator caspases (e.g., caspase-8), downstream effectors like PARP-1, or upstream regulatory molecules, effectively reduces neuronal apoptosis and ameliorates cognitive deficits following TBI. Therapeutic strategies targeting apoptosis are presented in Figure 1.

## 4. Molecular Mechanism of Necroptosis

Necroptosis, a regulated form of necrotic cell death first formally characterized by Degterev et al. in 2005, is morphologically defined by cellular swelling, organellar distension, plasma membrane rupture, and chromatin condensation [48,49]. Necroptosis is initiated through the activation of diverse signaling components, including cell surface death receptors, such as tumor necrosis factor receptor 1 (TNFR1) and Toll-like receptors (TLRs), as well as intracellular sensors of RNA and DNA [50]. The occurrence of necroptosis is contingent upon the sequential activation of receptor-interacting protein kinase 3 (RIPK3) and mixed lineage kinase-like pseudokinase (MLKL) [51]. RIPK3 activation occurs primarily through three distinct pathways. The first involves TNFR1-mediated activation of receptor-interacting protein kinase 1 (RIPK1), which then engages RIPK3 via mutual RIP (receptor-interacting protein) homology interaction motifs (RHIM) domain interactions [52]. Alternatively, TLR3 or TLR4 activation triggers the recruitment of RHIM-containing adaptor proteins that directly bind and activate RIPK3 [50]. A third pathway is initiated by the cytosolic nucleic acid sensor Z-dsDNA/dsRNA-binding protein 1 (ZBP1), which activates RIPK3 through its RHIM domain [53,54]. Subsequently, activated RIPK3 phosphorylates MLKL, which ultimately oligomerizes to form activated necrosomes that translocate to the plasma membrane, thereby triggering necroptosis [49].

## 5. Therapeutic Potential of Necroptosis in Post-TBI Cognitive Impairment

Research on necroptosis in cognitive impairment after TBI began in 2008. The study demonstrated that treatment of the necroptosis-specific inhibitor necrostatin-1 to TBI mice significantly reduced brain tissue injury volume and improved cognitive function, without decreasing apoptosis-related caspase-3 activity, thereby highlighting the specific contribution of necroptosis to post-TBI cognitive impairment [55]. Subsequent studies further explore the role of necroptosis in cognitive dysfunction following TBI. RIPK3, a key regulator of necroptosis, has been demonstrated to contribute to TBI-induced cognitive impairment, as evidenced by a study showing that RIPK3 knockout significantly improves spatial learning and memory in TBI mice [56]. Another study showed that neuron-specific deficiency of RIPK1 or RIPK3 significantly enhances cognitive performance of TBI mice, indicating that RIPK1 or RIPK3 serve as viable therapeutic targets for post-TBI cognitive impairment [57]. Consistent with this finding, evidence indicated that 2-(2-benzofuranyl)-2-imidazoline (2-BFI) attenuates necroptosis through downregulation of RIPK1 and RIPK3 expression, thereby ameliorating cognitive dysfunction in the TBI rat model [58]. Emerging evidence indicated that overexpression of charged multivesicular body protein 4B (CHMP4B) reduced RIPK3 levels, markedly diminished necroptosis, and promoted cognitive recovery in TBI mice [59]. Additionally, a pioneering study analyzing human brain samples provided direct evidence of necroptosis following TBI. The same investigation further demonstrated that both necrostatin-1 and melatonin reduced the levels of RIPK1 and RIPK3, inhibited necroptosis, and subsequently improved cognitive function post-TBI in rats [60].

**Figure 2:**
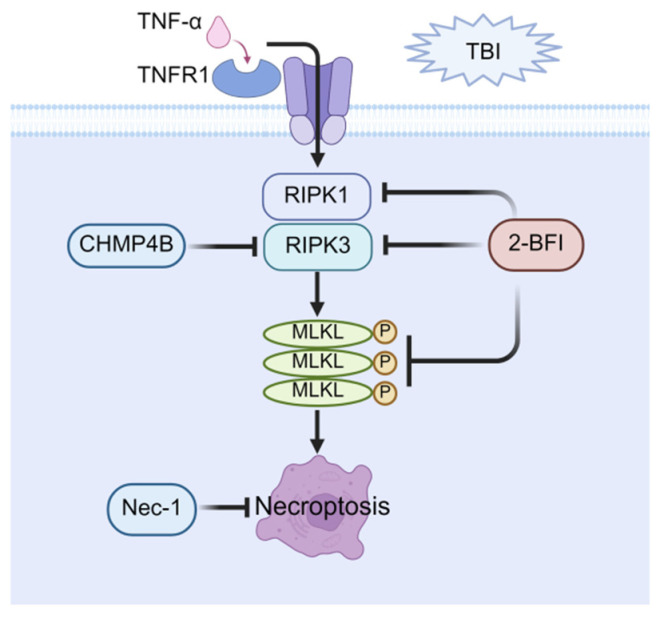
Therapeutic potential of necroptosis in post-TBI cognitive impairment. Necroptosis is mediated through RIPK1/RIPK3/MLKL activation, resulting in MLKL oligomerization, leading to necroptosis. Key pharmacological interventions targeting this pathway include: direct necroptosis inhibitors, Nec-1; and upstream pathway regulators (2-BFI: suppresses RIPK1/RIPK3/MLKL expression; CHMP4B: blocks RIPK3 activation). These strategies attenuate neuronal death and improve cognitive function after TBI.

In summary, targeted inhibition of key necroptotic signaling molecules, such as RIPK1, RIPK3, and MLKL, provides a promising therapeutic strategy to protect cognitive function after TBI. Given the established role of necroptosis in secondary injury progression, pharmacologic interventions like necrostatin-1 or novel small-molecule inhibitors offer significant potential for clinical translation, not only as acute neuroprotective treatments but also as preventive measures against long-term cognitive deficits. The major pathways related to necroptosis are illustrated in Figure 2.

## 6. Molecular Mechanism of Pyroptosis

Pyroptosis is an inflammatory form of cell death hallmarked by rupture of the plasma membrane, cell bubbling, and chromatin condensation [61,62]. In brief, the pyroptotic signaling cascade comprises two pathways: the canonical pathway mediated by caspase-1 and the non-canonical pathway driven by caspase-4, -5, or -11 [63]. The canonical pathway is the process by which various pathogen-associated molecular patterns (PAMPs) and damage-associated molecular patterns (DAMPs) are detected by cytoplasmic sensor proteins, such as absent in melanoma 2 (AIM2) [64]. Activated sensor proteins recruit and activate caspase-1 through the caspase recruitment domain (CARD)-containing adaptor apoptosis-associated speck-like protein, thereby assembling the NOD-like receptor family members 3 (NLRP3) inflammasome [65,66]. Subsequently, activated caspase-1 cleaves gasdermin D (GSDMD) while concurrently driving maturation of IL-1β and IL-18. Cleaved GSDMD liberates the N-terminal pore-forming domain (PFD), which inserts into the plasma membrane, oligomerizes, and forms transmembrane pores that execute pyroptosis [67]. Conversely, in the noncanonical pathway, bacterial lipopolysaccharide (LPS) directly activates caspase-11 in mice (or caspase-4/5 in humans), which then cleaves GSDMD to perforate the plasma membrane and trigger pyroptosis [68].

## 7. Therapeutic Potential of Pyroptosis in Post-TBI Cognitive Impairment

The association between pyroptosis and post-TBI cognitive impairment was recognized relatively recently. Studies have confirmed the upregulation of NLRP3 inflammasomes and caspase-1 in the cerebral cortex after TBI, even following mild injury [69,70]. Furthermore, genetic ablation of caspase-1 was shown to suppress pyroptosis and restore cognitive function in murine TBI models [71]. Building on this observation, subsequent work treated TBI mice with the caspase-1 inhibitor Ac-YVAD-cmk and confirmed that pharmacological blockade of caspase-1 attenuates pyroptosis and promotes cognitive recovery, underscoring the therapeutic potential of targeting caspase-1 for the treatment of post-TBI cognitive impairment [72]. At the execution level, caspase-1-cleaved GSDMD executes pyroptotic cell lysis. A study has shown that rhein attenuates post-TBI neurological deficits by down-regulating caspase-1 and GSDMD, leading to marked cognitive improvement [73]. Similarly, more recent research has confirmed that knocking out GSDMD in TBI model mice significantly elevates synaptic protein expression, thereby protecting cognition-related neural circuits and suggesting GSDMD as a promising therapeutic target [74].

Moreover, the NLRP3 inflammasome serves as a central regulatory hub within the molecular mechanisms governing pyroptosis. A study developed JC124, a small molecule that specifically targets NLRP3 inflammasomes. The research confirmed that JC124 can effectively inhibit NLRP3 activation and reduce neuronal degeneration and lesion volume in TBI [75]. This confirms the therapeutic potential of NLRP3-mediated pyroptosis in TBI and prompts multiple interventions targeting NLRP3 to alleviate post-TBI cognitive impairment. First, oridonin, the primary active component of the Chinese herbal medicine Rabdosia rubescens, has been reported to improve cognitive function in TBI mice by inhibiting NLRP3 inflammasome activation [76]. Similarly, dexmedetomidine has been shown to improve cognitive function in the rat TBI model by inhibiting NLRP3 inflammasome-mediated pyroptosis [77]. Consistently, several indirect pathways can also achieve similar effects by inhibiting the NLRP3 inflammasome. For example, blocking high mobility group box 1 (HMGB1) can prevent prolonged NLRP3 activation, significantly improving long-term potentiation (LTP) and memory function in TBI mice [78]. NIMA-related kinase 7 (NEK7) serves as a critical regulator of NLRP3 inflammasome activation, and its downregulation in TBI mice attenuates NLRP3 inflammasome activation, reduces neuronal damage, and ameliorates cognitive dysfunction post-TBI [79]. Additionally, a recent study has demonstrated that knockdown of galectin-3 inhibits the NLRP3 inflammasome, thereby mitigating pyroptosis and ameliorating neuronal damage and cognitive deficits post-TBI [80].

Non-pharmacological interventions also provide therapeutic benefits through modulation of pyroptosis pathways. In one investigation, transcranial pulsed current stimulation (tPCS) promoted orexin-A release, thereby inhibiting NLRP3 inflammasome formation and activation. This suppression reduced ROS production, decreased pyroptosis, and improved cognitive recovery in murine TBI models [81]. Separately, vagus nerve stimulation (VNS) has been shown to attenuate NLRP3 inflammasome activation and limit the release of cleaved caspase-1, ASC, and GSDMD. Through this modulation of neuroinflammatory cascades, VNS exerts neuroprotective effects and preserves cognitive function in mild TBI models [82].

**Figure 3:**
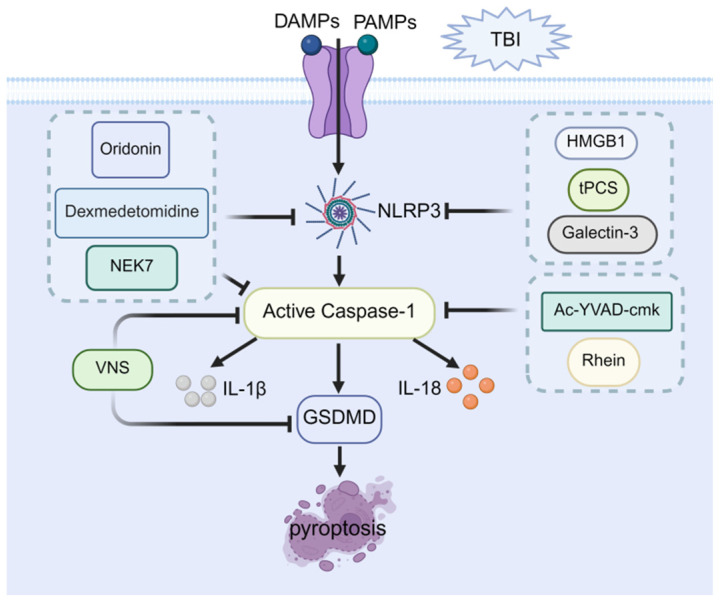
Therapeutic potential of pyroptosis in post-TBI cognitive impairment. Pyroptosis proceeds through NLRP3 inflammasome assembly and caspase-1 activation, which cleaves gasdermin D (GSDMD) to induce membrane pore formation and promote the maturation and release of IL-1β and IL-18. Pharmacological interventions alleviate neuroinflammation and cognitive impairment through direct targeting of NLRP3 inflammasome inhibition (e.g., by downregulating HMGB1 or galectin-3, and via transcranial Pulsed Current Stimulation (tPCS)); direct targeting of caspase-1 inhibition (using Ac-YVAD-cmk or Rhein); concurrent targeting of both NLRP3 and caspase-1 (via Dexmedetomidine or oridoninor and by downregulating NEK7); and multi-target agents such as Vagus Nerve Stimulation (VNS) (inhibiting NLRP3 and GSDMD).

Taken together, the aforementioned studies suggested that targeting key molecules involved in pyroptosis, particularly NLRP3, may represent a highly valuable and potentially therapeutic strategy for ameliorating cognitive dysfunction following TBI. Intervention strategies for pyroptosis are schematized in Figure 3.

## 8. Molecular Mechanism of Ferroptosis

Ferroptosis, a distinct iron-dependent form of regulated cell death first described by Dixon et al., is morphologically characterized by the preservation of plasma membrane integrity, normal nuclear morphology, mitochondrial shrinkage, rupture of the outer mitochondrial membrane, and reduction or loss of mitochondrial cristae [83]. Ferroptosis is primarily driven by iron overload and disruption of the redox balance [84]. On the one hand, transferrin (Tf) and transferrin receptor (TfR) form Tf–TfR complexes that facilitate the transport of Fe^3+^ into cells, where it is reduced to Fe^2+^. Any disturbance of iron homeostasis or an increase in free Fe^2+^ induces intracellular iron accumulation [85]. The accumulated iron, via the Fenton reaction, generates excessive reactive oxygen species (ROS) that trigger ferroptosis [86]. On the other hand, the SLC7A11/SLC3A2 complex constitutes the cystine (Cys_2_)-glutamate antiporter system Xc^−^. This Xc^−^ imports Cys_2_ to support glutathione (GSH) synthesis, and the GSH-dependent enzyme glutathione peroxidase 4 (GPX4) reduces toxic lipid peroxides to harmless alcohols [87,88]. Consequently, inhibition of Xc^−^, depletion of GSH, or suppression of GPX4 activity leads to lipid peroxide accumulation, thereby inducing ferroptosis [89].

## 9. Therapeutic Potential of Ferroptosis in Post-TBI Cognitive Impairment

Since the introduction of ferroptosis, numerous studies have revealed a robust link between iron overload and TBI. Magnetic resonance imaging (MRI) studies have revealed iron deposition in the thalamic region of TBI mice, which is further supported by a significant elevation in serum total iron levels observed as early as day 1 post-TBI [90,91,92]. Notably, ferroptosis can be induced even after mild TBI [93]. Consistent with these findings, Ji-Yao Jiang et al. reported iron accumulation, reduced GPX4 activity, and elevated ROS levels in TBI mice, with TEM further revealing mitochondrial atrophy—a hallmark of ferroptosis. Crucially, treatment with the ferroptosis-specific inhibitor Fer-1 attenuated iron deposition and neuronal death, while concurrently rescuing cognitive deficits post-TBI [94]. These findings suggest that ferroptosis serves as a critical driver of post-TBI cognitive impairment, thereby laying a solid foundation for the development of therapeutic strategies.

GPX4 serves as a central regulator of ferroptosis and confers neuroprotection [95]. Based on a study, the overexpression of GPX4 in the hippocampus of TBI mice reversed ferroptosis and synaptic damage, thereby markedly mitigating cognitive deficits, positioning GPX4 as a promising therapeutic target [96]. Netrin-1, a secretory laminin implicated in nerve regeneration, has been reported to activate the UNC5B/Nrf2 pathway, thereby upregulating GPX4 expression, suppressing lipid peroxidation, mitigating neuronal loss, and ultimately ameliorating spatial learning and memory impairment in TBI mice [97]. Furthermore, methyltransferase-like 3 (METTL3) elevated GPX4 levels by enhancing its mRNA stability, thereby suppressing ferroptosis and ameliorating cognitive impairment post-TBI [98]. Together, these findings consolidate the rationale for targeting GPX4 in cognitive impairment post-TBI therapy.

Certain therapeutic interventions attenuate ferroptosis by suppressing lipid peroxidation and intracellular iron accumulation through the modulation of core regulatory pathways. Specifically, these interventions downregulate TfR-mediated iron uptake, upregulate the SLC7A11 subunit of system Xc^−^, and enhance GPX4 expression, thereby attenuating neuronal damage and promoting cognitive recovery post-TBI. Anacardic acid (AA) and Fer-1 exert comparable neuroprotective effects against cognitive impairment post-TBI by mitigating ferroptosis through a concerted mechanism involving TfR downregulation and GPX4 upregulation [99]. Similarly, by downregulating TfR levels, inhibiting lipid peroxidation, and upregulating GPX4 expression, ruxolitinib mitigates neuronal oxidative damage, thereby enhancing spatial learning and memory in TBI mice [100]. In addition, aminophylline (AMP) markedly improves spatial cognition in TBI mice by downregulating microRNA-128-3p, which enhances SLC7A11 expression and consequently reduces lipid peroxidation [101]. Likewise, fisetin, a naturally occurring flavonoid, inhibits ferroptosis by elevating the expression of GPX4 and SLC7A11, thereby suppressing lipid peroxidation and iron accumulation, maintaining hippocampal neuronal integrity, and ultimately enhancing spatial memory after TBI [102]. Beyond pharmacological approaches, non-pharmacological interventions such as lifestyle modifications and physical therapies also have demonstrated considerable therapeutic potential. For example, median nerve stimulation (MNS) has been shown to upregulate GPX4, alleviate oxidative stress, inhibit ferroptosis, and ameliorate cognitive deficits in TBI rats [103]. Also, a study has demonstrated that moderate-intensity treadmill exercise downregulates TfR expression and upregulates GPX4, rescues ferroptosis, and thereby attenuates cognitive impairment post-TBI [104]. Furthermore, intermittent fasting has been shown to upregulate GPX4 expression, inhibit ferroptosis, and ameliorate cognitive deficits post-TBI, suggesting another promising therapeutic strategy for similar applications [105].

Acyl-CoA synthetase long-chain family members (ACSLs) are central to lipid metabolism and play a critical regulatory role in the process of ferroptosis [106,107]. Recent studies have demonstrated that modulation of ACSL can ameliorate ferroptosis-related cognitive impairment following TBI. For example, a study reported that prokineticin-2 (Prok2) overexpression attenuates neuronal degeneration and cognitive deficits by inhibiting ferroptosis, a mechanism mediated through the accelerated degradation of ACSL4 and a consequent reduction in lipid peroxidation substrates [108]. Similarly, a study has demonstrated that the downregulation of tumor necrosis factor-α-induced protein 3 (TNFAIP3) impairs the degradation of ACSL3, thereby suppressing lipid peroxidation and neuronal ferroptosis, which ultimately alleviates cognitive deficits induced by TBI [109].

**Figure 4:**
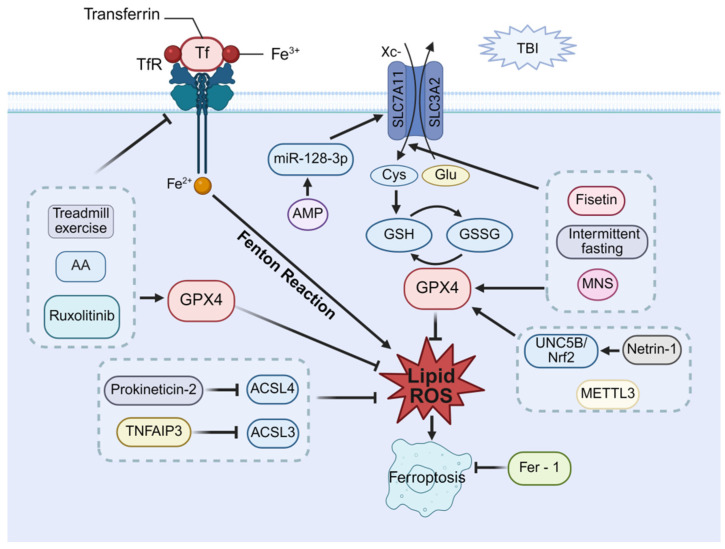
Therapeutic potential of ferroptosis in post-TBI cognitive impairment. Ferroptosis proceeds through two primary mechanisms: (1) dysregulated iron metabolism via TF/TfR, leading to Fe^2+^ accumulation and Fenton reaction-mediated lipid peroxidation, and (2) impaired system Xc^−^-GSH-GPX4 antioxidant axis, resulting in unchecked lipid peroxide accumulation. Therapeutic strategies to inhibit ferroptosis and improve cognitive function include modulation of iron metabolism (via moderate intensity of treadmill exercise, asiatic acid (AA), or Ruxolitinib, which inhibit transferrin receptor and upregulate GPX4); activation of system Xc^−^ (via AMP through the miR-128-3p/SLC7A11 axis, intermittent fasting and median nerve stimulation (MNS), or Fisetin by upregulating SLC7A11 and GPX4); enhancement of the GPX4 axis (via Netrin-1 activating the UNC5B/Nrf2/GPX4 pathway or METTL3); and suppression of lipid peroxidation (via Prokineticin-2 and TNFAIP3, which inhibit ACSL4/3 to reduce lipid peroxides).

In terms of ferroptosis, these collective findings underscore the therapeutic potential of targeting its pathways to ameliorate post-TBI cognitive deficits. Elucidating these mechanisms further provides a rationale for developing novel TBI therapeutic interventions, as summarized in Figure 4.

## 10. Molecular Mechanism of Cuproptosis

In 2022, Tsvetkov et al first proposed a copper-dependent mode of cell death, coining the term cuproptosis and describing its cytomorphology by mitochondrial condensation, rupture of the plasma membrane, endoplasmic reticulum damage, and chromatin condensation [110]. Notably, cuproptosis is not blocked by inhibitors of other known RCD, underscoring its mechanistic uniqueness. In essence, the disruption of copper homeostasis elevates intracellular copper levels, which initiates cuproptosis. In this process, copper carriers facilitate the import of Cu^2+^ into mitochondria, and this Cu^2+^ is then reduced to Cu^+^ by ferredoxin-1 (FDX1) [111]. Critically, the elevated Cu^+^ directly targets the acyl-carboxylase subunits dihydrolipoamide S-acetyltransferase (DLAT) and dihydrolipoamide S-succinyltransferase (DLST) within the tricarboxylic acid (TCA) cycle. This targeting promotes their oligomerization into insoluble aggregates, thereby disrupting normal TCA cycle metabolism. At the same time, excess copper destabilizes and degrades Fe-S cluster proteins [112]. The combined insult, TCA-cycle collapse plus Fe-S loss, generates proteotoxic stress that upregulates heat shock protein 70 (HSP70) and triggers cuproptosis [110].

## 11. Therapeutic Potential of Cuproptosis in Post-TBI Cognitive Impairment

**Figure 5:**
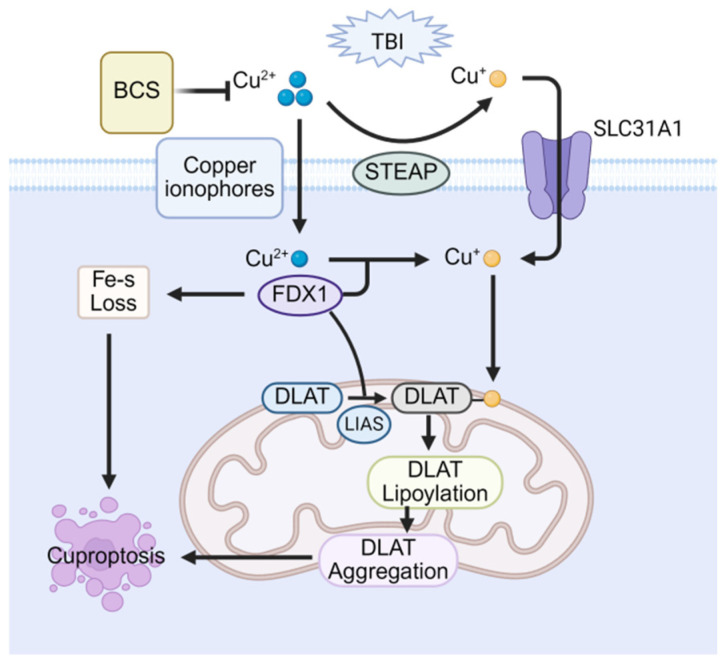
Therapeutic potential of cuproptosis in post-TBI cognitive impairment. Copper overload triggers cuproptosis via mitochondrial FDX1-mediated reduction of Cu^2+^ to Cu^+^, inducing lipoylated protein (e.g., DLAT) aggregation and Fe–S cluster protein loss. The copper chelator BCS attenuates cognitive impairment by sequestering excess copper and inhibiting this process.

Even before the term cuproptosis was introduced, numerous studies had already reported a correlation between copper overload and mild TBI [113]. Initially, two studies by Portbury et al. confirmed increased copper concentrations in the ipsilateral cerebral cortex of TBI mouse models. This increase was specifically observed in the region adjacent to the impact zone [91,114]. Furthermore, a separate study using PET/CT imaging assessed copper ion uptake and demonstrated significantly higher cortical copper levels in the TBI-injured group [115]. These findings substantiate the phenomenon of copper overload following TBI. However, cuproptosis is a newly recognized pathway, so data on its role in post-TBI cognitive impairment remain scarce and fragmentary. A study demonstrated that elevated copper in the brains of copper-exposed mice triggers a canonical cuproptosis signature. Excess copper further impaired synaptic function, which resulted in significant cognitive dysfunction. Conversely, the copper-selective chelator Bathocuproinedisulfonic acid (BCS) alleviated cuproptosis, underscoring cuproptosis as one of the key drivers of TBI-associated cognitive decline [116]. As the role of cuproptosis in post-TBI cognitive impairment becomes increasingly elucidated (Figure 5), there is hope for utilizing mechanisms of cuproptosis to treat memory and learning deficits, albeit further exploration remains warranted.

## 12. Interaction Between RCDs in Post-TBI Cognitive Impairment

TBI activates multiple RCD pathways, which do not function in isolation but instead form a highly interdependent regulatory network. Post-TBI insults, such as glutamate excitotoxicity, oxidative stress, and mitochondrial dysfunction, simultaneously engage multiple RCD mechanisms, amplifying neuronal loss and cognitive decline [117]. For example, oxidative stress induced by glutamate excitotoxicity activates cylindromatosis, which promotes necroptosis through RIPK1/RIPK3 complex formation while also enhancing mitochondrial AIF release to induce apoptosis [118]. In addition, iron accumulation, exacerbated by oxidative stress, triggers ferroptosis by decreasing GSH and GPX4 activity and generating ROS via the Fenton reaction. This oxidative burst activates the NLRP3 inflammasome, triggering pyroptosis and amplifying apoptosis through caspase-3 [119]. These findings establish a crucial role for synergistic interactions among RCD pathways in post-TBI cognitive impairment. Notably, key molecules within individual RCD pathways exhibit cross-pathway regulatory capacity. For instance, RIPK3, a central mediator of necroptosis in post-TBI cognitive impairment, not only facilitates necroptosis but also promotes apoptosis through caspase-8 and caspase-3 activation [56]. Furthermore, RIPK3 deficiency has been shown to redirect cell death signaling from pyroptosis toward ferroptosis through the 15-Lipoxygenase/phosphatidylethanolamine-binding protein 1 (15LOX/PEBP1) complex, highlighting the intricate crosstalk among RCD modalities [120].

Importantly, these RCD pathways exhibit distinct temporal dynamics following TBI. In the acute phase, ferroptosis predominates, characterized by rapid iron accumulation and lipid peroxidation. In contrast, apoptosis and pyroptosis markers, such as caspase-3 and NLRP3, show delayed activation, suggesting that ROS from iron-induced ferroptosis trigger subsequent RCD pathways [121]. During the chronic phase, necroptosis becomes dominant, with sustained ROS production from hemorrhage-derived iron accumulation perpetuating RIPK1/RIPK3 activation. Critically, genetic deletion of RIPK1/RIPK3 significantly reduced chronic lesion expansion by approximately 80%, underscoring the key role of necroptosis in chronic pathology [57].

The complexity and temporal dynamics of the post-TBI RCD network highlight the limitations of single-target therapies and underscore the need for multi-pathway, phase-adjusted interventions. Necrostatin-1, previously established as a necroptosis inhibitor, has also been shown to mitigate apoptosis in the context of post-TBI cognitive impairment [122]. Similarly, angiotensin-converting enzyme 2 (ACE2) has been identified as a key cross-regulatory node linking the renin-angiotensin system and the RCD network. Acute administration of recombinant human ACE2 curbed ferroptotic cascade amplification, whereas chronic delivery of the ACE2 activator diethylisopropylamine (DIZE) sustained ACE2 activity while concurrently blocking necroptosis, apoptosis, and inflammation-driven RCD amplification, exemplifying a phased, multi-pathway therapeutic strategy [123].

Overall, future TBI treatment strategies should emphasize multi-target and phase-specific interventions. By precisely targeting dominant RCD pathways at different disease stages, such approaches may achieve comprehensive regulation of the RCD network, thereby more effectively improving cognitive impairment after TBI.

## 13. Challenges and Prospects

Targeting RCD pathways represents a promising therapeutic strategy for cognitive impairment following TBI. However, its clinical translation faces several critical barriers. A primary challenge lies in the limited translational validity of preclinical models. While animal studies typically employ standardized injuries with single mechanisms, human TBI involves complex secondary pathologies such as hemorrhage, inflammatory cascades, and multi-organ damage. This substantial heterogeneity impedes the clinical replication of neuroprotective effects observed with RCD inhibitors in animal studies [124]. Furthermore, the blood–brain barrier (BBB) constitutes a major obstacle by restricting brain delivery of most RCD-targeting therapeutics. The dynamic evolution of BBB permeability during TBI progression—from acute disruption that facilitates drug entry to subsequent repair that restricts access—further complicates drug pharmacokinetics and challenges the development of sustained-release formulations [125].

To address these limitations, nanocarrier systems offer promising solutions through two complementary strategies. The first employs multitargeting nanoparticles to concurrently address several injury mechanisms, providing broader therapeutic efficacy than conventional drugs with single mechanisms. The second utilizes adaptive nanoparticles that respond to BBB changes, ensuring effective drug delivery during both acute and recovery phases of TBI to maintain therapeutic concentrations in the brain [126]. These integrated strategies align effectively with the complex pathophysiology of human TBI and establish a solid foundation for translating RCD-targeted therapies into clinical practice.

Human TBI research also confronts distinct challenges in clinical translation. Considerable patient heterogeneity in age, injury mechanisms, and severity leads to diverse RCD activation profiles, complicating the development of standardized treatments. Cognitive assessment is further constrained by the inherent subjectivity of conventional scales and the prolonged interval between initial pathology and observable symptomatic decline [127]. To overcome these issues, future research should increasingly integrate network pharmacology and artificial intelligence (AI). Network pharmacology was employed to identify potential therapeutic targets of Danshen-Chuanxiong-Honghua for cognitive impairment following TBI [128]. AI-driven analytics can identify patient subgroups with heightened responsiveness to specific RCD inhibitors—for instance, apolipoprotein E4 carriers or patients with elevated necroptosis activity—thereby advancing stratified precision medicine [129]. Additionally, machine learning models can dynamically correlate evolving RCD biomarker patterns with long-term cognitive outcomes [130].

Looking forward, integrated strategies combining nanomaterial-based delivery platforms, network pharmacology-enabled multitarget screening, and AI-guided patient stratification offer considerable promise for overcoming existing translational barriers in TBI-related cognitive impairment.

## 14. Conclusions

Cognitive impairment following TBI substantially diminishes patients’ quality of life, and the limited efficacy of existing treatments underscores the need for novel therapeutic strategies. This review systematically outlines key targets and intervention approaches across major RCD pathways, apoptosis, necroptosis, pyroptosis, ferroptosis, and cuproptosis (Table 1). While targeting the interconnected RCD network holds transformative potential, clinical translation faces challenges, including species differences, drug delivery limitations, and assessment standardization. Future research should focus on three pivotal directions: nanotechnology-enhanced drug delivery, network pharmacology for multi-target screening, and AI-driven precision medicine. Integrating RCD mechanism insights with innovative bioengineering approaches will be crucial for developing effective therapies to alleviate post-TBI cognitive impairment.

## Figures and Tables

**Table 1:** RCD in the post-TBI cognitive impairment model.

RCD Forms	Therapeutic Strategies	Models	Target Regulation	Regulation Mechanism	Behavior Test
Apoptosis	Z-DEVD-fmk [29]	Rat	Caspase-3 ↓	-	MWM: latency ↓
	INO-1001 [31]	Mice	AIF ↓	PARP-1 ↓	MWM: latency to hidden platform ↓, dwell time spent in target quadrant ↑
	Azithromycin [34]	Rat	Caspase-3 ↓, Bcl-2 ↑, BAX ↓	-	MWM: escape latency ↓, The time spent in target quadrant ↑, RT: fall off time ↑, OFT: number of boxes crossed ↑, NOR: time spent exploring objects ↑
	Lupeol [35]	Mice	BAX ↓, Cytc release ↓, Caspase-3 ↓	-	MWM: mean escape latency ↓, the time spent in the target quadrant ↑, Y-maze: spontaneous alteration ↑
	Edaravone [36]	Rat	Caspase-3 ↓, Bcl-2 ↑, BAX ↓	BDNF/TrkB ↑	MWM: escape latency ↓, the time spent in the target quadrant ↑
	PFT-α [37]	Rat	Caspase-3 ↓, Bcl-2 ↑, BAX ↓	P53 ↓	NOR: discrimination index ↑, OFT: moving time ↑
	COG1410 [38]	Mice	Caspase-3 ↓, Bcl-2 ↑, BAX ↓	Akt/CREB/BDNF ↑	MWM: latency ↓, target quadrant time ↑, OFT: center time ↑
	ZL006 [39]	Mice	Caspase-3 ↓, Bcl-2 ↑, BAX ↓	Interaction between nNOS and PSD95 ↓	MWM: latency ↓, the time to the target ↑, The number of crossings ↑, traveled distances ↑
	HS38 [40]	Mice	Caspase-3 ↓	ZIPK/DEDD ↓	OFT: the total distance traveled ↑, the number of entries to central zone ↑, MWM: latency ↓, the count of platform crossings ↑
	Mild hypothermia [41]	Rat	Caspase-3 ↓, Bcl-2 ↑	-	MWM: latency ↓, the number of platform crossings ↑, the time stayed in the target quadrant ↑
	Hyperbaric Oxygen Therapy [42]	Rat	Caspase-3 ↓	-	MWM: latency to platform ↓, RT: latency to fall ↑
Necroptosis	Necrostatin-1 [55]	Mice	Necroptosis ↓		MWM: latency ↓
	2-BFI [58]	Rat	RIPK1 ↓, RIPK3 ↓, MLKL ↓	-	Neurological score ↑
	Upregulate CHMP4B [59]	Mice	RIPK3 ↓		MWM: latency ↓, spent in the target quadrant ↑, the number of times crossed the target platform ↑, RT: latency to fall ↑
Pyroptosis	Ac-YVADcmk [72]	Mice	Caspase-1 ↓	-	MWM: escape latency ↓, time spent in the goal quadrant ↑, NOR: the index of exploring time ↑
	Oridonin [76]	Mice	NLRP3 ↓, Caspase-1 ↓	-	RT: latency to fall ↑
	Dexmedetomidine [77]	Rat	NLRP3 ↓, Caspase-1 ↓	-	MWM: latency ↓
	Rhein [73]	Mice	Caspase-1 ↓	-	RT: latency to fall ↑
	Downregulate HMGB1 [78]	Mice	NLRP3 ↓	-	T-maze test: alternation rate ↑ NOR: discrimination index ↑
	Downregulate NEK7 [79]	Mice	NLRP3 ↓, Caspase-1 ↓	-	RT: latency to fall ↑, OFT: distance traveled ↑
	Downregulate galectin-3 [80]	Mice	NLRP3 ↓	-	MWM: escape latency ↓, the frequency of the mice crossing the location of the platform ↑, the time spent in target quadrant ↑, Y-maze test: spontaneous alterations ↑
	Transcranial pulsed current stimulation (tPCS) [81]	Mice	NLRP3 ↓	-	Beam-balance tests: foot slips ↓, NOR: discrimination index ↑
	Vagus nerve stimulation(VNS) [82]	Rat	Caspase-1 ↓, GSDMD ↓	-	MWM: escape latency ↓, time spent in the target quadrant ↑, the number of platform crossings ↑
Ferroptosis	Fer-1 [94]	Mice	Ferroptosis ↓	-	MWM: latency ↓, Beam walk test: foot falls ↓
	Upregulate Netrin-1 [97]	Mice	GPX4 ↑	UNC5B/Nrf 2 ↑	MWM: latency ↓
	Upregulate METTL3 [98]	Mice	GPX4 ↑	-	MWM: latency ↓
	Ruxolitinib [100]	Mice	GPX4 ↑, TfR1 ↓	-	MWM: latency ↓, crossing number ↑
	Anacardic acid (AA) [99]	Rat	GPX4 ↑, TfR1 ↓	-	NOR: recognition index ↑, MWM: times of crossing ↑, time in the target quadrant ↑
	Aminophylline (AMP) [101]	Mice	SLC7A11 ↑	MicroRNA-128-3p ↓	MWM: latency ↓, the frequency of crossing quadrant ↑ RT: latency to fall ↑
	Fisetin [102]	Mice	GPX 4 ↑, SLC7A11 ↑	-	MWM: latency ↓, time spent in the target quadrant ↑
	Median nerve stimulation (MNS) [103]	Rat	GPX 4 ↑, SLC7A11 ↑	Nrf2/GPX 4 ↑	OFT: number of activities ↑, average speed ↑, resting times ↓
	Moderate intensity of treadmill exercise [104]	Mice	GPX4 ↑, TfR1 ↓	-	MWM: the number of crossings over the platform ↑, escape latency ↓ OFT: Time spent in the central field ↑ Three-Chamber Social Test: social novelty index ↑
	Intermittent fasting [105]	Mice	GPX4 ↑, SLC7A11 ↑	-	MWM: escape latency ↓, number of platform crossings ↑ NOR: exploration time ↑
	Upregulate prokineticin-2 [108]	Mice	-	ACSL4 ↓	MWM: latency to platform ↓
	TNFAIP3 [109]	Mice	-	ACSL3 ↓	MWM: latency to platform ↓, platform crossover number ↑
Cuproptosis	BCS [116]	Mice	Copper	-	MWM: escape latency ↓

↑: upregulated; ↓: downregulated; MWM: Morris water maze; OFT: open field test; NOR: novel object recognition test; RT: rotarod test.

## Data Availability

No new data were created or analyzed in this study. Data sharing is not applicable to this article.
